# A Novel Method for Assessing and Training Everyday Functional Skills

**DOI:** 10.1093/geroni/igaa057.1513

**Published:** 2020-12-16

**Authors:** Sara Czaja, Philp Harvey, Peter Kallestrup

**Affiliations:** 1 Weill Cornell Medicine, New York, New York, United States; 2 University of Miami Miller School of Medicine, Miami, Florida, United States; 3 i-Function, Miami, Florida, United States

## Abstract

Older adults, especially those with cognitive impairments often experience difficulty performing everyday tasks such as medication management, which threatens independence. Thus, there is an interest in developing treatment approaches for those who are experiencing or at risk for cognitive problems. Cognitive remediation training (CRT) programs have shown to be effective in improving cognitive abilities, but there is limited evidence to suggest that CRT results in everyday task performance gains. This presentation will discuss findings from a trial evaluating an innovative computer -based functional skills assessment and skills training program (CFSAT), which includes ecologically valid simulations of everyday tasks (e.g., shopping, money and medication management). The sample includes non-cognitively impaired (NC) older adults (n=50) and those with mild cognitive impairment (MCI) (n = 40), ranging in age from 60 -86 years (M= 73.10; SD = 6.06), is primarily female (90%), and ethnically diverse (69% minority). Participants were randomized into the CFSAT condition or a CFSAT + CRT condition. Performance data includes real time measures of accuracy, response time and efficiency. The findings indicate that the assessment component of the CFSAT program differentiated between the NC and MCI groups at the baseline assessment. Both NC and MCI participants demonstrated improvements in performance following training across all tasks; though the MCI participants required more training. Participants who received CFSAT + CRT training demonstrated increased efficiency in skill acquisition. The results indicate that the CFSAT program is an efficacious tool for assessing and training functional performance in both non-cognitively and cognitively impaired older adults.

